# A Retrospective Review of the Prognostic Value of ALDH-1, Bmi-1 and Nanog Stem Cell Markers in Esophageal Squamous Cell Carcinoma

**DOI:** 10.1371/journal.pone.0105676

**Published:** 2014-08-22

**Authors:** Cheng-Cheng Hwang, Shin Nieh, Chien-Hong Lai, Chien-Sheng Tsai, Liang-Che Chang, Chung-Ching Hua, Wen-Ying Chi, Hui-Ping Chien, Chih-Wei Wang, Siu-Cheung Chan, Tsan-Yu Hsieh, Jim-Ray Chen

**Affiliations:** 1 Department of Pathology, Chang Gung Memorial Hospital, Keelung, Taiwan; 2 Department of Pathology and Graduate Institute of Pathology, National Defense Medical Center & Tri-Service General Hospital, Taipei, Taiwan; 3 Division of Hematology-Oncology, Department of Internal Medicine, Chang Gung Memorial Hospital, Keelung, Taiwan; 4 Department of Radiation Oncology, Chang Gung Memorial Hospital, Keelung, Taiwan; 5 Division of Pulmonary, Critical Care and Sleep Medicine, Chang Gung Memorial Hospital, Keelung, Taiwan; 6 Department of Diagnostic Radiology, Chang Gung Memorial Hospital, Keelung, Taiwan; 7 Department of Anatomic Pathology, Chang Gung Memorial Hospital, Tao-Yuan, Taiwan; The Chinese University of Hong Kong, Hong Kong

## Abstract

Stem cell markers are upregulated in various cancers and have potential as prognostic indicators. The objective of this study was to determine the expression of three stem cell markers, aldehyde dehydrogenase 1 (ALDH-1), B cell-specific Moloney murine leukemia virus integration site 1 (Bmi-1), and Nanog, in esophageal squamous cell carcinoma (ESCC) tissues. Immunohistochemistry was used to measure the expression of ALDH-1, Bmi-1, and Nanog in ESCC tissues from 41 patients who received pre-operative chemoradiation. We evaluated the relationship between expression of these markers, and clinicopathological features, tumor regression grade (TRG), and 5-year overall survival (OS). There were no significant associations of ALDH-1 or Bmi-1 expression with age, gender, clinical stage, and treatments (*p*>0.05). However, patients with Nanog-positive tumors were significantly older than those whose tumors were Nanog-negative (*p* = 0.033). TRG after treatment was significantly associated with expression of ALDH-1 (*p* = 0.001), Bmi-1 (*p* = 0.004), and Nanog (*p*<0.001). Although OS was significantly better in patients with low TRGs (*p* = 0.001), there were no significant correlations between ALDH-1, Bmi-1, or Nanog with OS. Expression of ALDH-1, Bmi-1, and Nanog correlated with TRG, but not OS. Further large studies are necessary to fully elucidate the prognostic value of these stem cell markers for ESCC patients.

## Introduction

Esopahgeal adenocarcinoma (EAC) and esophageal squamous cell carcinoma (ESCC) are the 2 major histological types of esophageal cancer [1]. EAC is more common in Western countries, but ESCC is more common in East Asia [2]. In China, ESCC is the fourth-leading cause of cancer-associated death [3]. Despite improvements in surgery, chemotherapy, and radiotherapy, the 5-year overall survival remains poor, in part because patients typically have advanced-stage cancer at diagnosis [4]. Thus, in addition to prevention strategies, which focus on modification of risk factors associated with ESCC such as alcohol consumption and cigarette smoking [5], identification of prognostic markers of ESCC may also help to reduce the mortality associated with this cancer.

The levels of serum anti-p53 antibodies and C-reactive protein predict the response to therapy in patients with recurrent esophageal cancer [6]. In addition, the phosphorylation level of mammalian target of rapamycin (mTOR) is associated with response to chemoradiotherapy and overall disease-free survival in ESCC patients [7]. Furthermore, 2 mesenchymal markers of the epithelial mesenchymal transition (EMT), vimentin and fibronectin, are indicators of poor prognosis [4]. The EMT enhances cancer invasion and metastasis, in part because cells develop stem cell-like properties [8].

Cancer stem cells have the capacity of self-renewal, and are thought to be responsible for the generation of multiple cell lineages that are characteristic of many tumors [9]–[10]. Cancer stem cells have roles in tumorigenesis and resistance to therapy, so the identification of markers for cancer stem cell may provide important information regarding patient prognosis and response to therapy [11]–[12]. For example, previous research indicated that adenosine triphosphate-binding cassette superfamily G member 2 (ABCG2) expression was associated with ESCC patient survival [13] and Notch1 expression was associated with greater pathological grade and shorter OS in ESCC patients [14].

Identification of markers associated with ESCC may be useful for determining prognosis and response to therapy. Previous research indicated that the expression of 3 cancer stem cell markers, aldehyde dehydrogenase 1 (ALDH-1), B cell-specific Moloney murine leukemia virus integration site 1 (Bmi-1), and Nanog, are associated with esophageal cancer [15]–[17]. ALDH-1 is one of a family of enzymes that catalyze the oxidation of aldehydes to carboxylic acids; Bmi-1 is a polycomb ring finger oncogene that regulates p16 and p19; and Nanog is a homeobox transcription factor that has elevated expression in stem cells and some solid tumors. The present study used immunohistochemistry to investigate the expression of these 3 genes in human ESCC tissues and examined the relationship of their expression with clinicopathological characteristics and prognosis.

## Material and Methods

### Sample and data collection

The records of 41 ESCC patients who underwent preoperative chemoradiation therapy and radical esophagectomy/curative surgery from 2000 to 2012 at Keelung Chang Gung Memorial Hospital of Chang Gung Medical Foundation were retrospectively reviewed. Patient information, including gender, age, and clinicopathologic characteristics, was obtained from the Taiwan Cancer Registry, a national database. ESCC tissue samples were obtained from all participants for immunohistochemical analysis. The enrolled patients included 39 males and 2 females, and the median age was 54 years (range, 37–78 years). Histological analysis indicated that 2 patients (4.9%) had well-differentiated tumors, 27 patients (65.9%) had moderately differentiated tumors, and 12 patients (29.3%) had poorly differentiated tumors. The median follow-up time was 13.0 months (range 0.3–57.4 months). The duration of overall survival (OS) began at the day of surgery and ended at last follow-up or death.

Diagnosis was confirmed by pathological examination of hematoxylin and eosin staining results as described previously [18]. There were 5 tumor regression grades (TRGs), ranging from complete tumor response (TRG I) to no regressive changes within the tumor (TRG V) as previously described [19]. Specifically, TRG I was characterized by the absence of residual cancer and fibrosis throughout the esophageal wall; TRG II by the presence of rare residual cancer cells scattered through the fibrosis; TRG III by an increased number of residual cancer cells with predominant fibrosis; TRG IV by residual cancer outgrowing fibrosis; and TRG V by no regressive changes [19]. This study was approved by the institutional review board of the Keelung Chang Gung Memorial Hospital of CGMF, and written informed consent was obtained from all participants.

### Immunohistochemical staining

**Figure 1:**
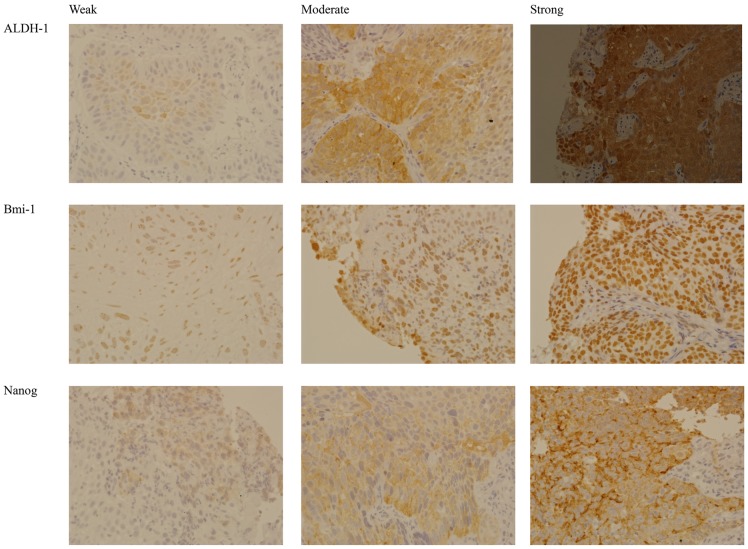
Representative images of cancer stem cell markers (top: ALDH1, middle: Bmi-1, bottom: Nanog) with different stain intensities (weak, moderate, strong).

Tissues were embedded in paraffin and sectioned. After deparaffinization, they were hydrated and incubated with an epitope retrieval solution (10 mM citric acid, pH 6.0) at 95°C for 40 min. After incubation with 0.3% H_2_O_2_ to inactivate endogenous peroxidase, nonspecific binding was inhibited by incubation with 5% normal serum in PBS at room temperature for 30 min. The sections were next incubated with primary antibodies specific for ALDH-1, Bmi-1, and Nanog (Epitomics, Burlingame CA, USA) at 4°C overnight. The sections were next incubated with biotinylated secondary antibodies (Lab Vision, Kalamazoo, MI, USA) at room temperature for 10 min, and then with streptavidin peroxidase (Lab Vision) at room temperature. Visualization was done with DAB following the manufacturer's instructions (DaKo, Carpinteria, CA, USA). After sections were counterstained with hematoxylin, they were air-dried and observed under a microscope (E600, Nikon, Tokyo, Japan). All cells of each section were observed. The stain intensity was graded as negative (no expression), weak (fewer than one-third positive cells), moderate (one-third to two-thirds positive cells), or strong (more than two-thirds positive cells). Figure 1 shows representative images of the different stain intensities of each marker. Two pathologists who were blinded to the clinical data performed all histological evaluations. Inconsistent results were reviewed and discussed before making a final decision.

### Statistical Analysis

Due to the small sample size, continuous data were compared using the Mann-Whitney U test, and categorical variables were compared by Fisher's exact test. Continuous variables are presented as medians and interquartile ranges, and categorical data as numbers and percentages. Correlations between stem cell marker expression and tumor regression grade were performed using Kendall's correlation coefficient. Kaplan-Meier analysis and the log-rank test were used for analysis of cumulative survival curves. All statistical assessments were two-sided, and a *p*-value less than 0.05 was considered significant. Statistical analyses were performed with SPSS 15.0 Statistics Software (SPSS Inc., Chicago, IL, USA).

## Results

### Association of stem cell markers with patient characteristics

Table 1 shows that there were no significant correlations between expression of ALDH-1 or Bmi-1 with age, gender, clinical stage (*p*>0.05 for all). However, patients with Nanog-positive tumors were significantly older than those whose tumors were Nanog-negative (*p* = 0.033). Additional analysis and calculation of Kendall's rank correlation coefficients indicated that there were several significant positive correlations in the expression of these markers (ALDH-1 and Bmi-1: τ = 0.34, *p* = 0.014; ALDH-1 and Nanog: τ = 0.45, *p* = 0.001; Bmi-1 and Nanog: τ = 0.28, *p* = 0.040) (Table S1).

**Table 1:** Relationship between expression of stem cell markers and characteristics of 41 patients with ESCC.

	ALDH-1			Bmi-1			Nanog		
	Positive (n = 24)	Negative (n = 17)	*P*-value	Positive (n = 36)	Negative (n = 5)	*P*-value	Positive (n = 37)	Negative (n = 4)	*P*-value
**Age (years)^1^**	55 (51, 64)	51 (46, 59)	0.255	54 (47, 62)	56 (53, 61)	0.282	55 (50, 62)	46 (41, 50)	0.033
**Gender, n (%)^2^**			1.000			1.000			1.000
** Male**	23 (95.8)	16 (94.1)		34 (94.4)	5 (100)		35 (94.6)	4 (100)	
** Female**	1 (4.2)	1 (5.9)		2 (5.6)	0 (0.0)		2 (5.4)	0 (0.0)	
**Clinical stage, n(%)^2^**			0.212			0.435			0.621
** II**	2 (8.3)	5 (29.4)		6 (16.7)	1 (20.0)		7 (18.9)	0 (0.0)	
** III**	14 (58.3)	9 (52.9)		19 (52.8)	4 (80.0)		21 (56.8)	2 (50.0)	
** IV**	8 (33.3)	3 (17.6)		11 (16.7)	0 (0.0)		9 (24.3)	2 (50.0)	

Each number indicates median and interquartile range unless otherwise specified.

*P*-values are based on ^1^Mann-Whitney U test and ^2^Fisher's exact test.

### Association of stem cell markers with TRG and patient survival

**Figure 2:**
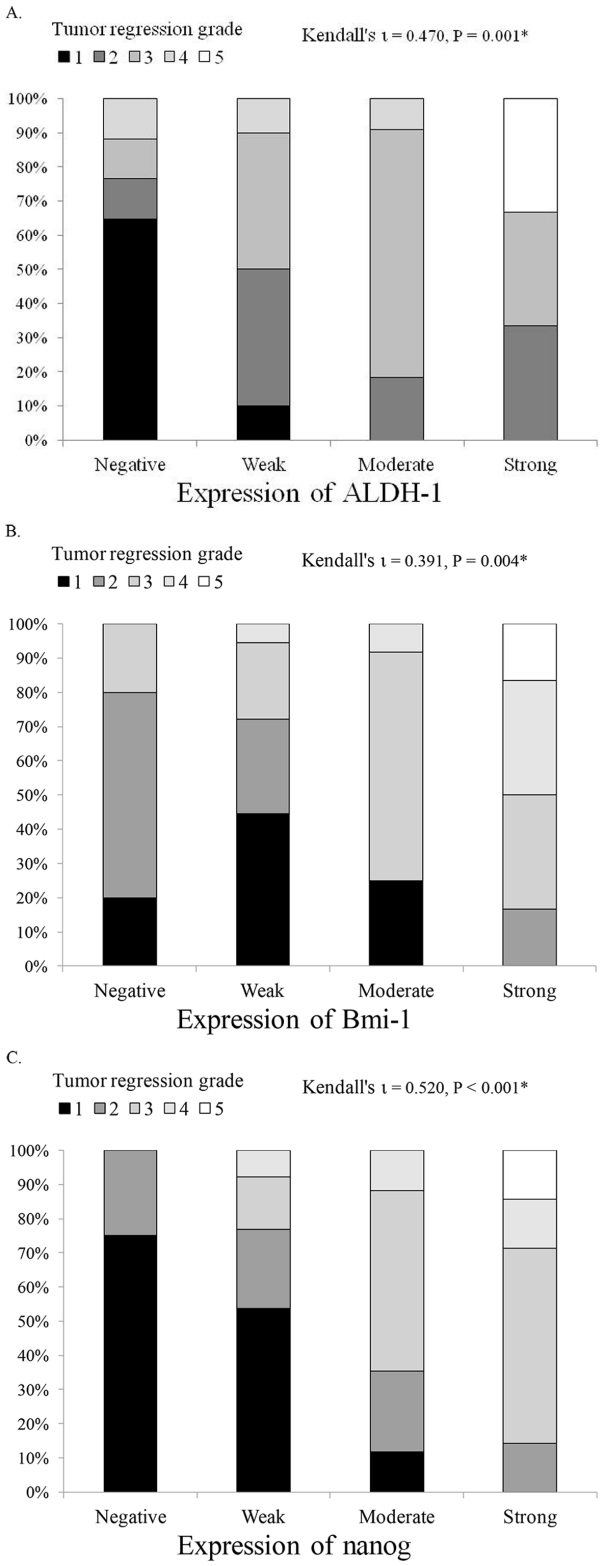
Relationship of expression of cancer stem cell markers (A: ALDH-1, B: Bmi-1, C: Nanog) with tumor regression grade. ^*^ *P*<0.01.

TRG was classified based on complete tumor response to therapy [19]. Figure 2 shows that TRG after treatment was significantly associated with greater expression of ALDH-1 (*p* = 0.001), Bmi-1 (*p* = 0.004), and Nanog (*p*<0.001).

**Figure 3:**
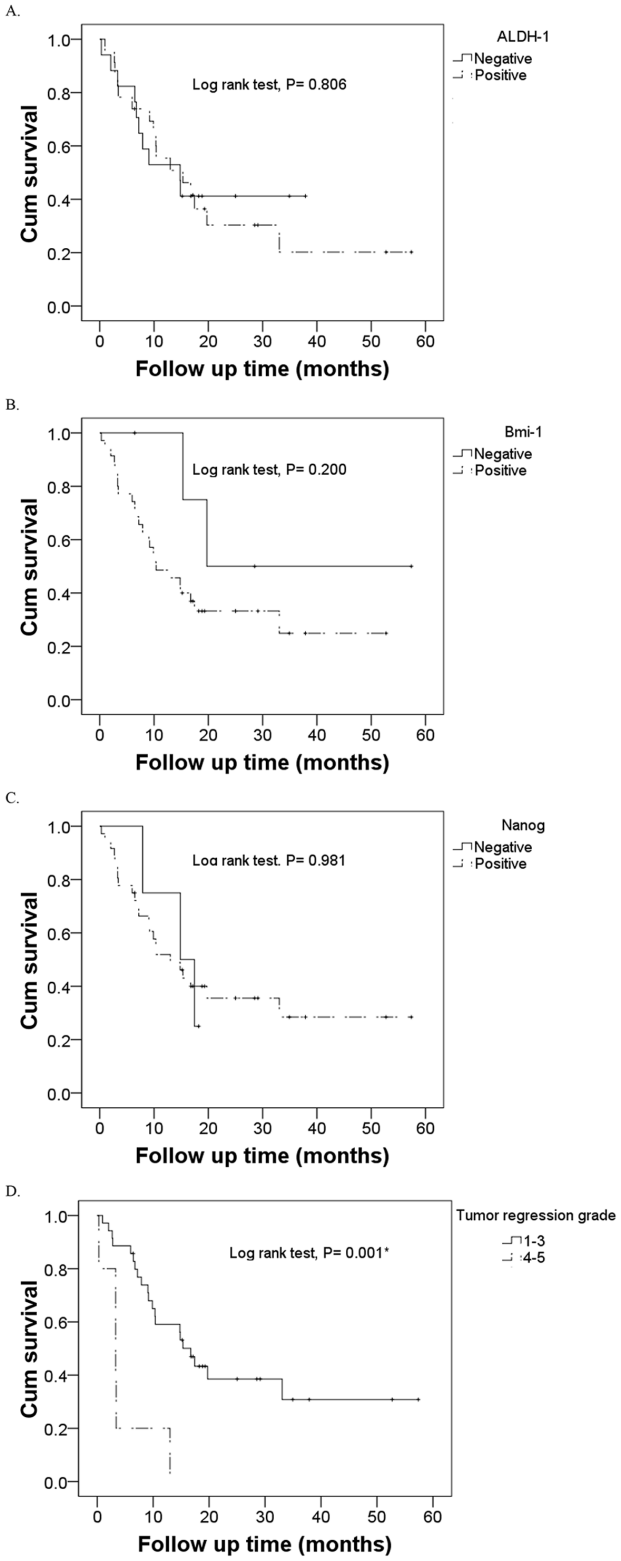
Kaplan-Meier analysis of the prognostic value of the expression of cancer stem cell markers (A: ALDH-1, B: Bmi-1, C: Nanog) and tumor regression grade (D) in prediction of overall survival. ^*^ *P*<0.001.

We also analyzed the association between the expression of the 3 stem cell markers with patient survival. By the end of the study, 15 patients had died from ESCC, 9 patients died from complications associated with ESCC, and 2 patients died from unknown causes. The median survival time was 14.8 months (95% CI, 8.8–20.8 months). Kaplan–Meier analysis and the log-rank tests show that there was no significant correlation between expression of any of the stem cell markers with OS (*p*>0.05). However, OS was significantly better in patients TRG 1–3 than in patients with TRG 4–5 (*p* = 0.001, Figure 3).

## Discussion

More than 50% of patients with esophageal cancer have metastases or unresectable tumors at diagnosis [20], and this largely explains their poor prognosis [4]. Therefore, identification of early diagnostic markers of esophageal cancer may be useful for improving prognosis and informing treatment decisions. Previous research reported that the expression of ALDH-1, Bmi-1, and Nanog were associated with ESCC and EAC [15]–[17], and this motivated our examination of the relationship between their expression and the clinicopathological features of patients with ESCC. Although Nanog expression was associated with age, there were no other significant correlations between expression of the 3 examined markers with age, gender, and clinical stage. However, elevated expression of each marker was associated with more advanced TRG after treatment. Although poor OS was associated with more advanced TRG, there were no significant correlations between expression of ALDH-1, Bmi-1, or Nanog with OS.

TNM staging, which considers tumor size, its spread to lymph nodes, and distant metastasis, is the most common staging system but a recent study highlighted several shortcomings of the 7^th^ edition regarding its prognostic value for patients receiving neoadjuvant chemoradiotherapy [21]. Thus, Weider et al. [22] suggested that assessments of early tumor response to chemoradiotherapy predicted esophageal cancer patient survival. Similarly, Mandard et al. [19] observed that tumor regression was a significant predictor of disease-free survival of esophageal cancer patients, consistent with the results of the present study, which indicated that advanced TRG was associated with poor OS. However, identification of prognostic markers that can be detected by noninvasive methods would enable clinicians to continuously monitor response to therapy. Cancer stem cells are important for tumorigenesis and therapy resistance, so analysis of cancer stem cell markers in tumors may provide important information on patient prognosis and response to therapy [11]–[12].

ALDH-1 has a role in stem cell differentiation by regulating retinal oxidation to retinoic acid [23], and its expression has been used as a marker for normal and cancer stem cells [24]. In Chinese patients with ESCC, nuclear expression of ALDH-1 is associated with poor histological differentiation, lymph node metastasis, TNM stage, and poor 5-year OS [15]. In the present study, ALDH-1 expression was associated with advanced TRG, but not with clinical stage or OS.

Bmi-1, a polycomb-group protein, regulates gene expression by chromatin remodeling, and control of axial patterning, cell proliferation, and senescence [25]–[26]. Expression of Bmi-1 is upregulated in many solid and hematologic cancers [27]. In addition, previous research reported associations between Bmi-1 expression and ESCC and EAC [17], [28]–[29]. Specifically, He et al. [28] reported an association between Bmi-1 overexpression and advanced pathological stage and poor OS and the presence of Bmi-1 autoantibodies correlated with tumor stage and lymph node metastasis [29]. However, Choy et al. [17] found no association between Bmi-1 expression and OS in patients with EAC or ESCC, consistent with the results of the present study.

The expression of Nanog, a homeobox transcription factor, correlates with cell differentiation [30] and resistance to therapy [31] in patients with breast cancer and with poor survival in patients with oral cancer [32]. Furthermore, a previous study reported that Nanog expression was associated with TNM stage and differentiation of ESCC [16]. The present study indicated that Nanog expression was associated with TRG, but not with clinical stage or OS.

The small sample size of the present study is a limitation. Thus, the findings of no associations between ALDH-1, Bmi-1, or Nanog expression with clinicopathological features or OS cannot be completely ruled out. A larger cohort study would also allow the analysis of the prognostic value of these markers on tumor subpopulations, as in Sudo et al. [4]. Alternatively, it remains possible that the selected stem cell markers may not be suitable for predicting the outcome of patients with ESCC. Therefore, larger studies are necessary. In addition, the present study did not perform survival analysis based on tumor node metastasis stage of ESCC.

## Conclusions

Examination of the expression of 3 cancer stem cell markers, ALDH-1, Bmi-1, and Nanog, in patients with ESCC indicated correlations with TRG but not OS. Further large studies are necessary to fully elucidate the prognostic value of these stem cell markers for ESCC patients.

## Supporting Information

Table S1 **Correlations in the expression of ALDH-1, Bmi-1, and Nanog in 41 patients with ESCC.**
(DOC)
