# Supplementary material for: A Retrospective Review of the Prognostic Value of ALDH-1, Bmi-1 and Nanog Stem Cell Markers in Esophageal Squamous Cell Carcinoma
Source: PLoS One. 2014 Aug 22;9(8):e105676. doi: 10.1371/journal.pone.0105676 (PMC4141830; doi:10.1371/journal.pone.0105676)
Supplement: Table S1 — Correlations in the expression of ALDH-1, Bmi-1, and Nanog in 41 patients with ESCC. (DOC) [file pone.0105676.s001.doc]

**Table S1.** Correlations in the expression of ALDH-1, Bmi-1, and Nanog in 41 patients with ESCC.

|  | **ALDH-1** | **Bmi-1** | **Nanog** |
| --- | --- | --- | --- |
| **ALDH-1** | 1.00 | 0.34^*^ | 0.45^*^ |
| **Bmi-1** |  | 1.00 | 0.28^*^ |
| **Nanog** |  |  | 1.00 |

* *P*<0.05 by Kendall’s correlation coefficient
